# Detection of IgE, IgG, IgA and IgM antibodies against raw and processed food antigens

**DOI:** 10.1186/1743-7075-6-22

**Published:** 2009-05-12

**Authors:** Aristo Vojdani

**Affiliations:** 1 822 S. Robertson Blvd, Ste. 812, Los Angeles, CA 90035, USA

## Abstract

**Background:**

Despite the first documented case of food allergy to cooked food in 1921 by Prausnitz and Kustner, all commercial food antigens are prepared from raw food. Furthermore, all IgE and IgG antibodies against dietary proteins offered by many clinical laboratories are measured against raw food antigens.

**Methods:**

We developed an enzyme-linked immunosorbent assay for the measurement of IgE, IgG, IgA and IgM antibodies against raw and processed food antigens. Sera with low or high reactivity to modified food antigens were subjected to myelin basic protein, oxidized low density lipoprotein, and advanced glycation end products (AGE) such as AGE-human serum albumin and AGE-hemoglobin.

**Results:**

Compared to raw food antigens, IgE antibodies showed a 3–8-fold increase against processed food antigens in 31% of the patients. Similarly, IgG, IgA and IgM antibodies against modified food antigens overall were found at much higher levels than antibody reactions against raw food antigens. Almost every tested serum with high levels of antibodies against modified food antigens showed very high levels of antibodies against myelin basic protein, oxidized low density lipoprotein, AGE-human serum albumin and AGE-hemoglobin.

**Conclusion:**

We conclude that the determination of food allergy, intolerance and sensitivity would be improved by testing IgE, IgG, IgA and IgM antibodies against both raw and processed food antigens. Antibodies against modified food antigens, by reacting with AGEs and tissue proteins, may cause perturbation in degenerative and autoimmune diseases such as diabetes, atherosclerosis, inflammation, autoimmunity, neurodegeneration and neuroautoimmunity.

## Background

Adverse reactions to foods in which the pathogenesis involves an immunological response to food components are appropriately called food-hypersensitivity reactions. This term is considered to be synonymous with "food allergy." This adverse immune reaction to food proteins affects many children and adults [1]. In a study using double-blind placebo-controlled food challenge, 39% of participants showed hypersensitivity to food antigens [2].

Based on clinical presentation and antibody response, immune-mediated adverse reactions to foods can be divided into immediate and delayed hypersensitivity reactions. Immediate reactions to food antigens are IgE-mediated and dependent on activation of mast cells in specific tissues, including the skin, respiratory tract, gastrointestinal, mucosal, and cardiovascular system [3-5].

The delayed immune reaction to food antigens are mediated by IgG, IgA and IgM. Unlike the immediate effects of IgE-mediated allergy, the IgG, IgM and IgA-mediated food allergy and intolerance reactions can take several days to appear. Therefore, levels of IgG, IgM and IgA antibodies in the blood against different food antigens have been used for demonstration of delayed food allergy and intolerance reactions [6,7].

Despite the first documented case of food allergy to cooked food in 1921 by Prausnitz and Kustner [8], all commercial food antigens are prepared from raw food. However, for demonstration of both immediate and delayed hypersensitivity to food, antibodies are measured against antigens prepared from raw food [9-11].

Processed foods and their ingredients are subjected to a variety of conditions, which may cause alterations in immunodominant epitopes, potentially affecting allergenic properties. This processing may destroy existing epitopes on a protein or may cause new ones to be formed (neoallergen formation) as a result of change in protein conformation. Neoallergen formation has been known for at least three decades [12]; it may be part of the reason why some individuals can tolerate a raw food or raw food ingredient but will react to the same food when it is processed. Studies have found neoallergens from pecans [13], wheat flour [14], roasted peanuts [15], lentil [16], almond, cashew nut and walnut [17], soybean [18,19], shrimp, scallop, tuna, egg, apple, plum, milk and potato [2,11,20-22].

The different types of food processing includes thermal as well as non-thermal treatments, and each type of process may have a different effect on epitopes. In evaluating allergen stability, then, the different effects of individual treatments must be considered carefully. Thermal processing may be done by dry heat (e.g. oven roasting, oil roasting, infra-red heating, ohmic heating) or wet heat (e.g. boiling, microwave cooking, pressure cooking, autoclaving, extrusion, blanching, steaming). Non-thermal treatments include irradiation, soaking, germination, milling, fermentation, high-pressure processing, dehulling and dehusking, and grinding. Processing may affect food in a manner that may induce the masking or unmasking of allergenic epitopes, thereby enhancing or reducing allergen recognition and potentially altering the allergenicity of the offending food [21]. In relation to common processing methods, including mechanical, enzymatic, heating, drying, peeling, pulping, blanching, mashing, pasteurization and multiple-treatment effects on the allergenicity of processed food antigens, all the published articles dealt with immediate hypersensitivity reaction which is IgE mediated. None of these articles dealt with delayed immune reaction to processed food antigens [1-22]. This sampling of articles also illustrates that in a majority of cases some of the technological processing treatments not only maintained their antigenicity and allergenicity but also induced the modification and introduction of neoantigens. Therefore, this study was designed to assess both IgE- and non-IgE-mediated hypersensitivity against both raw and processed food antigens. The measurements of IgE, IgG, IgA and IgM antibodies in blood against processed food antigens results in an enhancement in the detection of delayed food sensitivities. This would not be possible by merely measuring these same antibodies against antigens prepared from raw or unprocessed foods alone.

## Methods

Sera from 40 food-allergic individuals and 40 healthy subjects were obtained from DPC Inc., Los Angeles, CA, and Innovative Research Inc., Novi, MI. Food antigens were prepared from food products purchased from large supermarket chains to reflect American purchasing and accessibility of food products. Each advertised ingredient of the prepared, cooked and processed foods was carefully catalogued at the lab. Some examples of the raw and processed foods used for the extraction of antigens are shown in Table 1. Hemoglobin (Hb), human serum albumin (HSA) and myelin basic protein (MBP) were purchased from Sigma Aldrich, St. Louis, Missouri, USA.

**Table 1:** Examples of raw and processed foods used for antigenic extraction and measurement of IgE, IgG, IgM and IgA antibodies in blood

A – Raw Food	B – Processed Food	A – Raw Food	B – Processed Food
1. Apple	Apple Cider	26. Wheat (Semolina)	Pasta
2. Pork	Bacon	27. Peanut	Peanut, Roasted
3. Barley, Hops, Yeast	Beer	28. Pecan	Pecan, Roasted
4. Chicken Wing, Wheat, Soy	Buffalo Wing	29. Cucumber, Vinegar	Pickles
5. Wheat, Barley, Yeast, Soy, Sugar	Doughnut	30. Wheat, Milk, Cheese, Olives, Tomato, Spinach, Mushroom, Broccoli	Pizza
6. Wheat, Egg, Corn, Milk, Sugar	Cake	31. Corn, Butter	Popcorn
7. Wheat, Oat, Corn, Rice, Barley, Milk, Sugar	Cereal	32. Egg, Potato, Cucumber, Corn, Mustard Seed, Vinegar, Lemon	Potato Salad
8. Chicken, Wheat, Rice, Veg. Oil	Chicken Chow Mein	33. Pumpkin, Wheat, Egg, Corn, Milk, Soy	Pumpkin Pie
9. Chicken, Wheat, Veg. Oil	Chicken, Fried	34. Rice, Wheat, Peanut, Soy	Rice Cake
10. Chicken, Wheat, Orange, Veg. Oil	Chicken, Orange	35. Salmon	Salmon, Baked
11. Beans, Chili Powder, Tomato, Onion, Corn, Potato, Garlic	Chili (Vegetarian)	36. Salmon	Salmon, Fried
12. Coffee	Coffee, Roasted	37. Beef, Pork, Soy, Wheat	Sausage
13. Cranberry, Corn	Cranberry Sauce	38. Shrimp	Shrimp, Cooked
14. Egg, Raw	Egg, Cooked	39. Soybean	Soy Agglutinin
15. Egg, Assorted Vegs., Wheat, Soy	Egg Roll	40. Beef Filet Mignon	Steak, Filet Mignon
16. Corn Starch, Sugar Dextrose, Yellow #5, Yellow #6, Red #3, Red #40, Blue #1	Food Coloring	41. New York Strip	Steak, New York
17. Potato, Veg. Oil, Wheat, Milk	French Fries	42. Potato, Veg. Oil	Tater Tots
18. Beef, Onion, Seasoning	Hamburger	43. Soybean, Casein	Tofu
19. Beef, Pork, Turkey, Corn, Wheat	Hotdog	44. Tuna	Tuna, Canned
20. Milk, Corn, Cocoa, Gum, Strawberries, Sugar	Ice Cream	45. Tomato, Carrot, Celery, Beet, Parsley, Lettuce, Spinach	Vegetable Juice
21. Tomato, Corn, Onion, Garlic, Vinegar	Ketchup	46. Wheat	Wheat Germ Agglutinin
22. Lentil	Lentil, Boiled	47. Milk, Corn, Coconut, Palm Oil, Veg. Oil	Whipped Cream
23. Egg, Soybean Oil, Lemon, Vinegar	Mayonnaise	48. Whitefish	Whitefish, Baked
24. Mustard Seed, Turmeric, Paprika, Vinegar	Mustard	49. Whitefish	Whitefish, Fried
25. Peanut, Veg. Oil	Peanut Butter	50. Grape, Yeast	Wine

### Analytical method for identification and characterization of food antigens

Each raw or processed food was ground at 4°C using a food processor and extraction buffers and reagents, such as Coco buffer (0.55% NaHCO_3_, 1% NaCl), 0.1 M phosphate buffer saline pH 7.4, 70% ethanol, and cold acetone.

Each food was mixed in four different solvents and kept on the stirrer for 2 h at room temperature. After centrifugation at 2000 g for 15 minutes the liquid phase from each buffer and solvent was removed and dialysed against 0.01 M PBS using dialysis bags with a cutoff of 6,000. The pellet from the acetone was dissolved in 0.1 M PBS buffer and dialysed. Dialysis was repeated for three times in order to make sure that all small molecules are removed. After dialysis extracted antigens from the above conditions were combined, and protein concentrations were measured using a kit provided by Bio-Rad (Hercules, CA).

For quality control in separating the antigens from the foods, sodium dodecylsulfate (SDS) gel electrophoresis was performed. The electrophoretic methods included SDS acrylamide gel (gradient of 7–15%), and Western blot [23,24].

### Detection of IgE, IgG, IgA and IgM antibodies by Enzyme-Linked ImmunoSorbent Assay (ELISA)

Dietary proteins and peptides were dissolved in phosphate buffered saline (PBS) at a concentration of 1.0 mg/ml then diluted 1:100 in 0.1 M carbonate-bicarbonate buffer, pH 9.5. 100 μl of each antigen was added to duplicate wells of a polystyrene flat-bottom ELISA plate. Plates were incubated overnight at 4°C and then washed three times with 20 mM Tris-buffered saline (TBS) containing 0.05% Tween 20. After washing, the plates were coated with 200 μl of 1.5% bovine serum albumin (BSA) and 1.5% gelatin in TBS and then incubated for 2 hours at room temperature and then overnight at 4°C. When used in combination, BSA+gelatin results in a very low non-specific binding OD <0.1. After the overnight incubation, the BSA+gelatin was removed. Plates were washed three times with 20 mM TBS containing 0.05% Tween 20, dried and stored at 4°C.

Quality control was performed by the addition of serum with low, medium and high titers of antibodies against different food antigens. In addition, plates were studied for the detection of non-specific reaction by the addition of all reagents except serum. After the performance of quality control, the plates were kept at 4°C until used.

For IgE determination the sera were diluted 1:2 in serum diluent buffer (20 mM TBS containing 1% BSA, 0.05% Tween 20, and 0.01% sodium azide, while for IgG, IgA and IgM determination the sera were diluted 1:100 in the same buffer; 100 μl of diluted sera were then added to duplicate wells of microtiter plates and incubated for an appropriate length of time: 2 hours for IgG, IgA and IgM, and overnight for IgE at 4°C. A standard curve was constructed using serum with a titer of 1:128 for IgE against hydrolyzed milk (protein concentration 1 mg/mL). A different serum with a titer of 1:12,800 against wheat proteins (concentration 1 mg/mL) was used to construct standard curves for IgG, IgA and IgM antibodies using antigens prepared from cereal. Plates were washed 5 times, and then 100 μl of alkaline phosphatase labeled goat anti-human IgE, IgG, IgM or IgA F(ab')_2 _fragments (KPI, Gaithersburg, MD) at an optimal dilution of 1:400 – 1:2000 in 1% BSA-TBS were added to each well; plates were incubated for an additional 2 h at room temperature. After washing five times with TBS-Tween buffer, the enzyme reaction was started by adding 100 μl of paranitrophenylphosphate (PNPP) in 0.1 mL diethanolamine buffer containing 1 mM MgCl_2 _and sodium azide pH 9.8. The reaction was stopped 45 min later with 50 μl of 1 N NaOH. The optical density (OD) was read at 405 nm by means of a microtiter reader. To detect non-specific binding, several control wells containing all reagents except human serum were added and used in each assay. The OD for all control wells coated with different antigens first and then with BSA+gelatin was less than 0.1 or 2 EU. The ELISA units (EU) of IgE, IgG, IgA or IgM antibodies against specific food antigens was determined by plotting the antibody's concentration on the log scale against the OD on the linear scale for seven calibrators with a known titer of 4, 8, 16, 32, 64, 96 and 128. The EU was determined using a computer program with linear regression capability and the generation of a curve that best fits the data and the following formula:



For example, using this formula, optical densities of 0.17, 0.3, 0.5, 0.8, 1.4, 2.0 and 2.7 resulted in EUs of 4, 8, 16, 32, 64, 96 and 128 respectively.

### Preparation of Advance Glycation End product (AGE) proteins

AGE-hemoglobin (AGE-Hb) and AGE-human serum albumin (AGE-HSA) were prepared as described previously [25] and later modified [26]. Briefly, 0.5 g HSA or Hb was dissolved with 3.0 g D-glucose in 10 mL of 0.1 M phosphate buffer saline pH 7.4 containing 0.05% sodium azide. For controls Hb and HSA were dissolved in PBS without D-glucose. Each solution was deoxygenated with nitrogen, sterilized by ultrafiltration (0.2 μm filter) and incubated for 45 days at 37°C. The samples were then dialyzed against 0.1 M PBS and were separated on a gradient of 7–15% of acrylamide. In comparison to samples without glucose, shift in Hb and HSA band in samples containing D-glucose was used for confirmation of AGE-Hb and AGE-HSA formation.

### Measurement of IgG antibodies against AGE-Hb and AGE-HSA

Hb, HSA, AGE-Hb and AGE-HSA were diluted to 100 μg/mL, and similar to the above ELISA, 100 μl of each antigen was added to duplicate wells of microtiter plated followed by incubation, washing, coating with BSA-gelatin, and repeated washing. Eight different sera with severe reaction against modified food antigens and 8 healthy control sera with very mild reaction against processed food antigens were applied to duplicate wells coated with Hb, HSA, AGE-Hb or AGE-HSA. After incubation, washing, addition of anti-human IgG secondary antibody, repeated washing and substrate, color development was measured. Sera from individuals with a titer of 1:12,800 against boiled egg antigens were used to construct a standard curve and the calculation of antibody units against AGE-Hb or AGE-HSA.

For the determination of specific IgG antibodies against AGE-Hb and AGE-HSA ELISA values obtained against Hb or HSA alone were subtracted from the ELISA values of AGE-Hb or AGE-HSA.

### Measurement of IgG antibodies against myelin basic protein (MBP)

MBP IgG antibody was measured according to the previously described method [27].

### Measurement of IgG antibodies against oxidized low density lipoprotein (ox-LDL)

ox-LDL IgG antibody was measured by a kit manufactured by Biomedica Gruppe and distributed in the US by ALPCO Diagnostics, Salem, New Hampshire.

## Results

### Detection of IgE antibodies against raw and processed food antigens

**Figure 1:**
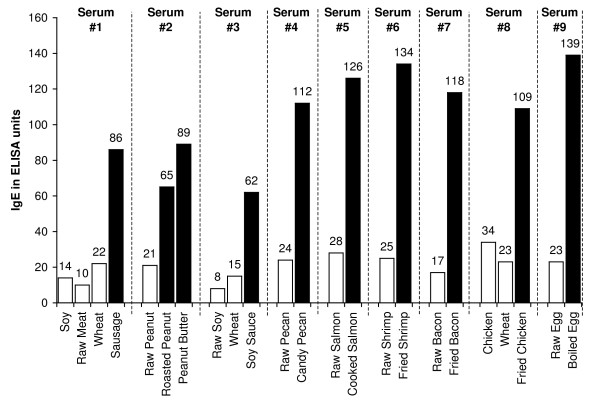
**Serum levels of IgE Antibodies against Raw (white square) vs Processed (black square) Food Antigens expressed by ELISA unit**. Measurement of IgE antibodies against different raw or crude ingredients versus the processed or cooked version of the foods in the sera of nine individuals with food allergy expressed in ELISA units.

Sera from 40 individuals with known type-1 allergic reaction to foods and sera from 40 healthy controls were screened for food-specific IgE antibodies by the Immulite DPC test system. 26 out of the 40 samples from the known allergic individuals and 3 out of the 40 samples from healthy control subjects that demonstrated IgE antibodies > 15 IU against five or more food antigens were selected for simultaneous measurement of IgE antibodies against raw and modified food antigens. These 29 samples with food-specific IgE antibodies > 15 IU were subjected to ELISA plates coated with raw and modified food antigens. 20 out of the 29 specimens showed very similar IgE antibody levels (± 20%) against different raw and modified food antigens. The other 9 samples (31%) showed 3–8-fold increase in IgE antibodies against modified food antigens as compared to the raw food antigens. For example, Sample #1 with OD of 0.32 or 10 EU of IgE against raw meat, OD of 0.41 or 14 EU against soy, and OD of 0.59 or 22 EU against wheat exhibited OD of 1.9 or 86 EU of IgE antibodies against antigens extracted from sausage. Sample #9 with OD of 0.6 had a level of 23 EU of IgE against raw egg, but had an OD of 3.2 or IgE level of 139 EU against boiled egg antigens (Figure 1). The data presented in Figure 1 clearly shows significant elevation in IgE antibodies against processed food in 9 serum samples from food-allergic individuals.

### Detection of IgG, IgA and IgM antibodies against raw and modified food antigens

All 80 sera specimens (40 with known IgE reaction to foods and 40 healthy controls) were subjected to ELISA plates coated with raw and modified food antigens for measurement of IgG-, IgA- and IgM-specific antibodies. Compared to sera from healthy controls, specimens with IgE reaction to food antibodies demonstrated elevations in IgG, IgA and IgM antibodies against both raw and modified food antigens. Overall this immune reaction against different modified food antigens was much higher than reaction against raw food antigens. Due to the high volume of data generated in this study, for clarity's sake only one patient's results are presented.

**Figure 2:**
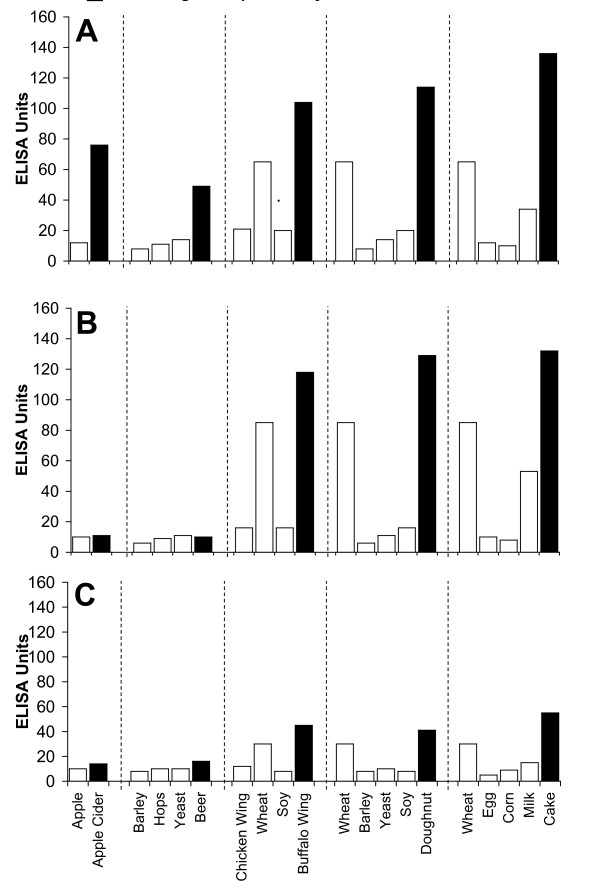
**Serum Levels of IgG (A), IgA (B), and IgM (C) against Raw (white square) vs Processed (black square) Food Antigens Expressed by ELISA Units**. Measurement of IgG, IgA and IgM antibodies against different raw or crude ingredients versus the processed or cooked versions of specific foods in the serum of a patient with high reactivity.

**Figure 3:**
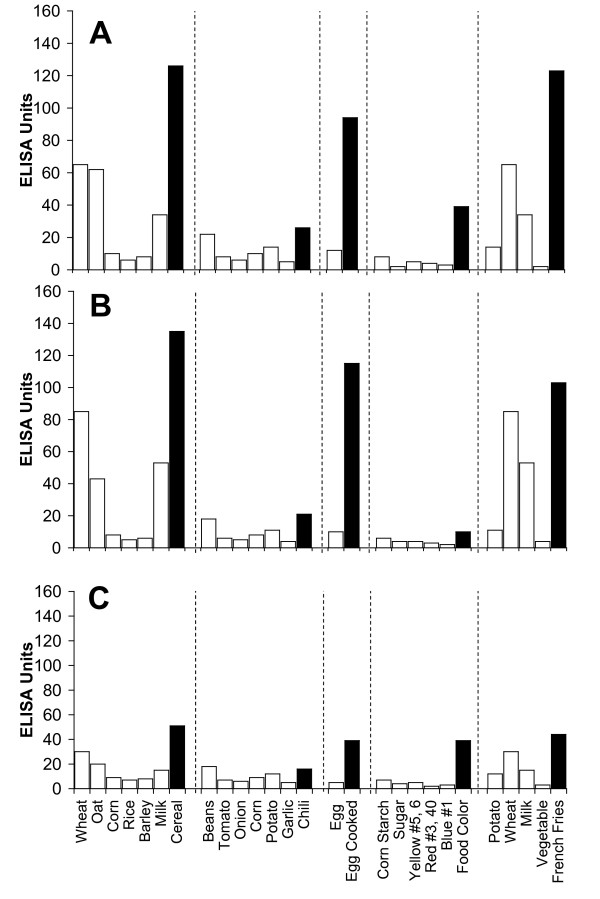
**Serum Levels of IgG (A), IgA (B), and IgM (C) against Raw (white square) vs Processed (black square) Food Antigens Expressed by ELISA Units**. Measurement of IgG, IgA and IgM antibodies against different raw or crude ingredients versus the processed or cooked versions of specific foods in the serum of a patient with high reactivity.

**Figure 4:**
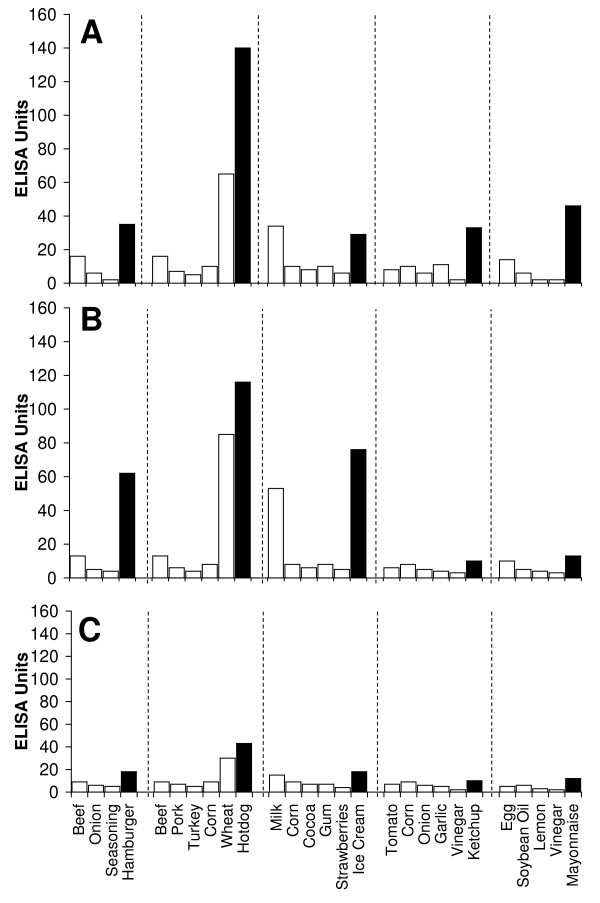
**Serum Levels of IgG (A), IgA (B), and IgM (C) against Raw (white square) vs Processed (black square) Food Antigens Expressed by ELISA Units**. Measurement of IgG, IgA and IgM antibodies against different raw or crude ingredients versus the processed or cooked versions of specific foods in the serum of a patient with high reactivity.

**Figure 5:**
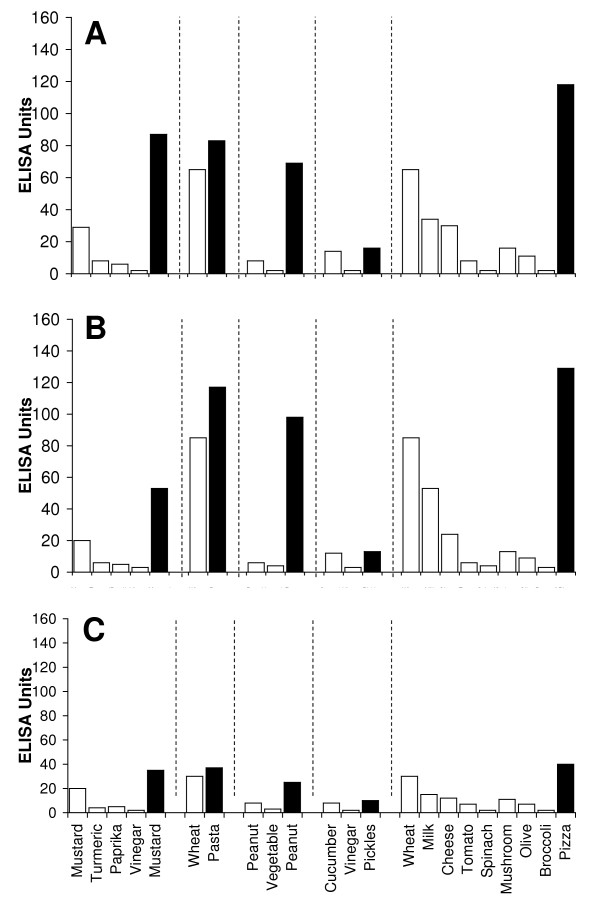
**Serum Levels of IgG (A), IgA (B), and IgM (C) against Raw (white square) vs Processed (black square) Food Antigens Expressed by ELISA Units**. Measurement of IgG, IgA and IgM antibodies against different raw or crude ingredients versus the processed or cooked versions of specific foods in the serum of a patient with high reactivity.

**Figure 6:**
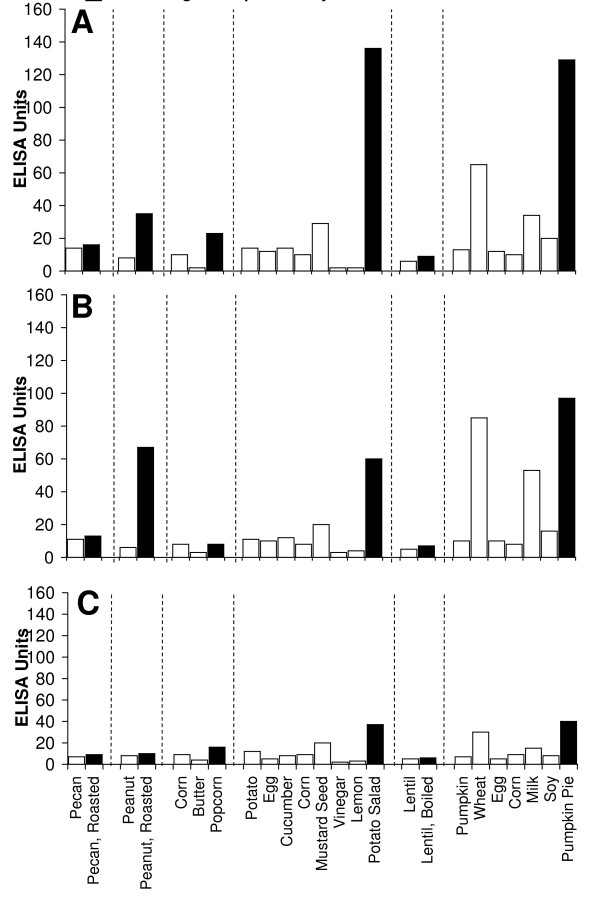
**Serum Levels of IgG (A), IgA (B), and IgM (C) against Raw (white square) vs Processed (black square) Food Antigens Expressed by ELISA Units**. Measurement of IgG, IgA and IgM antibodies against different raw or crude ingredients versus the processed or cooked versions of specific foods in the serum of a patient with high reactivity.

**Figure 7:**
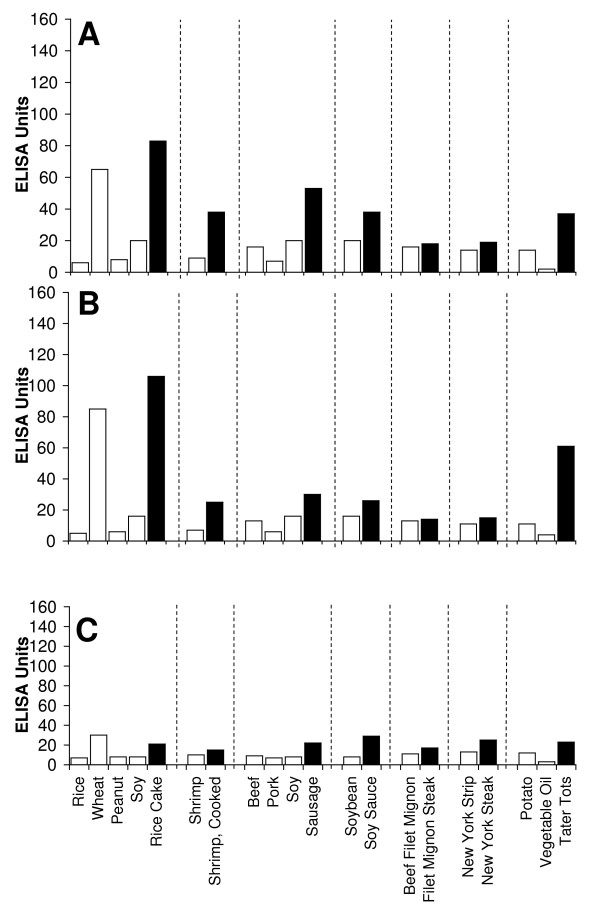
**Serum Levels of IgG (A), IgA (B), and IgM (C) against Raw (white square) vs Processed (black square) Food Antigens Expressed by ELISA Units**. Measurement of IgG, IgA and IgM antibodies against different raw or crude ingredients versus the processed or cooked versions of specific foods in the serum of a patient with high reactivity.

**Figure 8:**
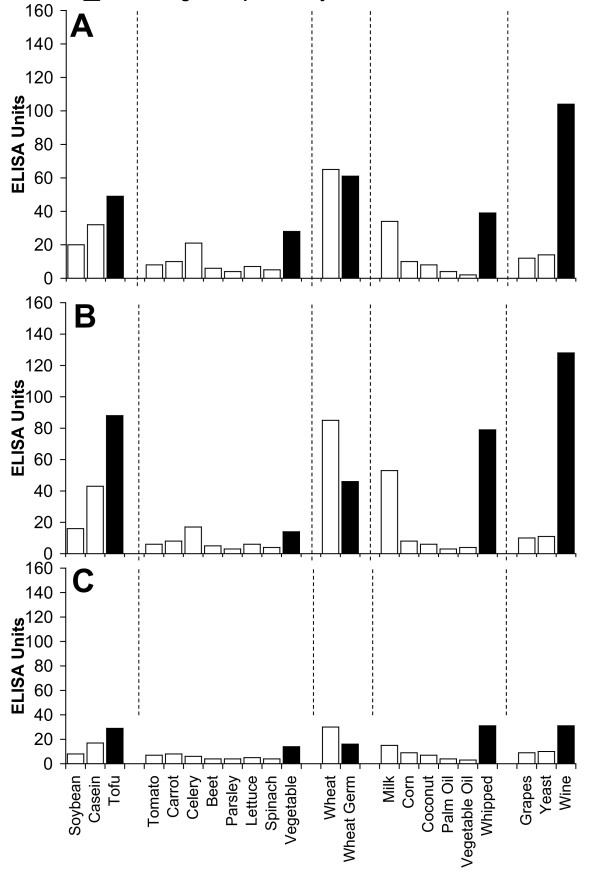
**Serum Levels of IgG (A), IgA (B), and IgM (C) against Raw (white square) vs Processed (black square) Food Antigens Expressed by ELISA Units**. Measurement of IgG, IgA and IgM antibodies against different raw or crude ingredients versus the processed or cooked versions of specific foods in the serum of a patient with high reactivity.

**Figure 9:**
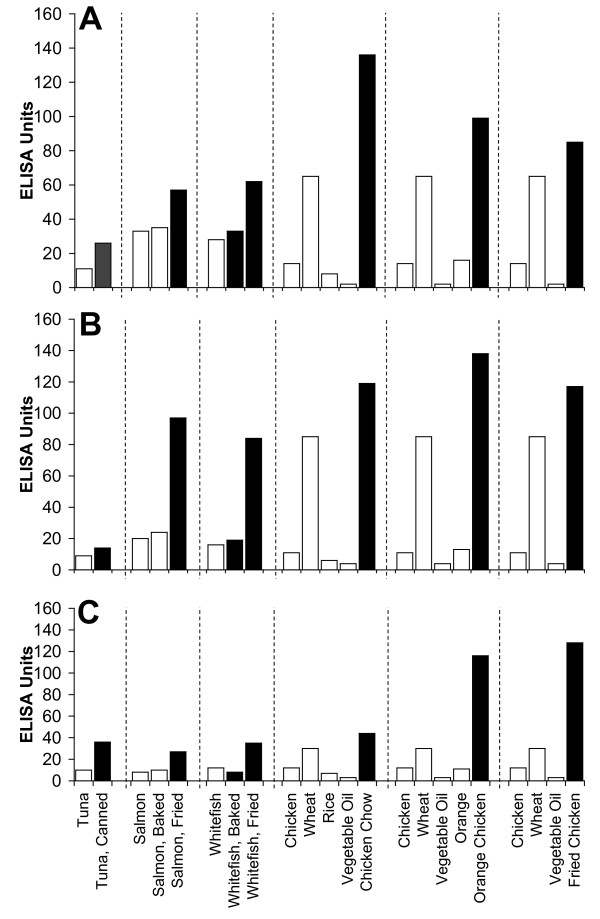
**Serum Levels of IgG (A), IgA (B), and IgM (C) against Raw (white square) vs Processed (black square) Food Antigens Expressed by ELISA Units**. Measurement of IgG, IgA and IgM antibodies against different raw or crude ingredients versus the processed or cooked versions of specific foods in the serum of a patient with high reactivity.

Figure 2, Figure 3, Figure 4, Figure 5, Figure 6, Figure 7, Figure 8, Figure 9A show the concentration of IgG antibodies in serum expressed by ELISA units against raw versus processed foods. Note in this example the high levels expressed against a majority of the processed or modified versions of the foods in comparison to their raw or crude forms. The results of 8 patients with high IgG reactivity against many modified food antigens were compared to 8 controls with low reactivity against these same modified antigens, as is summarized in Table 2. The total number of reactions >30 EU against modified food antigens in the 8 patients with high reactivity was 91 out of 360 determinations, while in the control group only 13 out of the same 360 determinations were >30 EU. The difference in reactivity against the same modified food antigens in these two groups was highly significant (p < 0.000001).

**Table 2:** Comparison of 8 patients with IgG reactivity >30 EU against many modified food antigens with 8 controls with low reactivity to few modified food antigens in relation to possible cross-reactivity with AGE-HSA, AGE-Hb, anti-ox-LDL, and MBP

	Sample #	No. of modified food antigens with IgG reactivity > 30 EU	IgG against AGE-HSA in EU	IgG against AGE-Hb in EU	IgG against anti-ox-LDL in mU	IgG against MBP in EU
8 patients with high reactivity to many modified food antigens	1	8/45	86	105	1642	118
	2	12/45	43	91	2050	136
	3	7/45	32	26	916	65
	4	16/45	120	111	2431	129
	5	11/45	61	23	1442	84
	6	9/45	25	39	683	27
	7	13/45	97	108	1216	120
	8	15/45	134	82	734	71

	Total	91/360	598	585	11,114	750

8 controls with low reactivity to few modified food antigens	9	1/45	12	7	265	15
	10	0/45	6	13	448	17
	11	2/45	19	22	1025	31
	12	4/45	15	26	683	18
	13	0/45	8	16	343	22
	14	1/45	63	46	205	75
	15	2/45	38	27	412	49
	16	3/45	24	13	157	95

		13/360	185	170	3538	322

P values		<0.000001	<0.005	<0.005	<0.005	<0.005

Figure 2, Figure 3, Figure 4, Figure 5, Figure 6, Figure 7, Figure 8, Figure 9B show the concentration of IgA antibodies in serum expressed by ELISA units against raw versus processed foods. Note in this example the high levels of IgA antibodies expressed against a majority of the processed or modified versions of the foods in comparison to their raw or crude forms. In addition, we observed a significant correlation between IgG and IgA antibodies against various raw and modified food antigens. Interestingly, IgG and IgA were detected against many raw and modified food antigens simultaneously, and the IgM level was much lower than the IgG and IgA antibodies (Figure 2, Figure 3, Figure 4, Figure 5, Figure 6, Figure 7, Figure 8, Figure 9).

### Interaction of IgG antibodies of sera recognizing modified food antigens with AGE-albumin, AGE-hemoglobin, oxidized low density lipoprotein and myelin basic protein

Eight different patients' sera (#s1–8) with high reactivity (8–16 out of 45 antigens) and eight healthy control sera (#s9–16) with low reactivity (0–4 out of 45 antigens) against 45 modified food antigens were selected for measurement of IgG against AGE-HSA, AGE-Hb, ox-LDL and MBP. Seven out of eight sera with IgG reactivity against modified food antigens showed significant elevation against AGE-HSA, AGE-Hb, ox-LDL and MBP (Table 2). For example, Sample #4 with IgG immune reaction against 16 out of 45 modified food antigens showed simultaneous elevation in antibodies against AGE-HSA (120 EU), AGE-Hb (111 EU), ox-LDL (2,431 mU) and MBP (129 EU). In comparison, only 1 (#14) out of 8 controls showed moderate elevation in IgG antibodies against these molecules. This individual showed IgG elevation against 1 out of 45 modified food antigens. Overall, the differences in IgG antibody levels against AGE-HSA, AGE-Hb, ox-LDL and MBP in 8 samples with high IgG antibodies against modified food antigens versus 8 samples with low IgG antibodies against modified food antigens were statistically very significant with p < 0.005. This simultaneous detection of IgG antibodies against modified food antigens and AGE-HSA, AGE-Hb, ox-LDL and MBP may indicate cross-reaction between modified food antigens and human tissue antigens modified by oxidation or glycation.

## Discussion

Delayed food sensitivity is associated with a multitude of disorders, such as multiple sclerosis, autism and rheumatoid arthritis, and affects an estimated 40% of the population. Patients presenting with clustered symtpoms of migraine, mood swings, fatigue, intestinal upset, joint pain, high blood pressure and attention problems are often found to have delayed immune reaction to food antigens [2,3,28]. Thus, it is vital to offer the medical community a sensitive method for the detection of food allergy and intolerance testing that is scientifically supported.

One reason traditional food sensitivity testing fails is that it does not reflect a true, real-world, contemporary, non-raw food diet. Widely-used food antigen testing uses only raw food isolates. Since the discovery of fire, fewer and fewer people consume a diet consisting solely of raw foods. As was shown in the introduction, researchers have demonstrated that chemical/molecular changes occur during food preparation, cooking and processing. In addition to altering the makeup of a single food, other changes to the food can occur when this food is combined with another during cooking and processing. Thus, a person who is allergic to ketchup may not have an immune reaction to a raw tomato. If tested using traditional food sensitivity assays, this patient would result negative for tomato, and the patient's problems would be unresolved [10,11,13,14,16,18,19,21,22,29-32]. Despite all of these findings documented in scientific literature and the demonstration of IgE-mediated reaction to many processed food antigens while not reacting to raw foods, almost all commercial laboratories measure IgE and IgG antibodies primarily against raw food antigens.

In this study we sought to prepare extracts from processed foods and compare them to extracts from raw food samples. Since all examples of new allergenicity to food antigens shown here and many others published in scientific journals deal only with IgE mediated or type-I allergic reaction, we decided to confirm these findings with raw and processed food antigens prepared in our laboratory. For example, in Serum #1 the assay detected 10 units of IgE against raw meat, 22 units of IgE against wheat antigens, 14 units of IgE against soy, and 86 units of IgE against wheat isolates prepared from sausage. Or in Serum #7, only 17 units of IgE were detected against raw bacon, but 134 units of IgE were detected against antigens prepared from fried bacon, which is part of a contemporary American diet (Figure 1). These results confirm an earlier study [14] that reported a case of food allergy to a wheat isolate used in sausage and pork pie, but without any allergic reaction to native wheat flour. This allergic reaction to wheat isolates was attributed to the induction of cryptic allergenic or the formation of new allergenic epitopes by technological and chemical processes [14]. Similarly, a case of contact urticaria has recently been attributed to hydrolyzed wheat in cosmetics combined with a generalized urticaria induced with the ingestion of sausages with lentils and a French cassoulet [14]. It was concluded that wheat isolates should be tested when a food allergy to finished food is suspected. In addition to IgE-mediated food sensitivity, delayed food sensitivity has become a growing concern for practitioners in many medical fields. In light of this we decided to extend this investigation to non-IgE-mediated antibodies produced against extracts prepared from modified foods purchased from supermarkets or restaurants (as shown in Table 1). This includes the measurement in duplicate of IgG, IgM and IgA in blood against modified food antigens. Only data from one out of eighty tested sera is presented in Figure 2, Figure 3, Figure 4, Figure 5, Figure 6, Figure 7, Figure 8, Figure 9. This and other data (not shown) clearly indicate that an individual may not show IgG, IgA and IgM antibodies against antigens prepared from raw food, but testing the same patient against antigens prepared from cooked food might result in a severe immune reaction.

To examine possible cross-reactivity and the clinical significance of these antibodies in inflammation and autoimmunities, we selected 8 sera from patients with significant reaction and 8 sera from healthy controls with no or very moderate reaction against modified food antigens and reacted them with AGE-HSA and AGE-Hb. The data presented in Table 2 shows that out of 8 samples with IgG antibodies >30 EU against 8–16 modified food antigens, 6 had IgG antibodies against AGE-HSA and 6 against AGE-Hb. In comparison healthy controls with low levels of antibodies against modified food antigens produced very low levels of antibodies against AGE-HSA and AGE-Hb. These results suggest that AGE formation through the reaction of protein amino groups with sugar may be an important chemical pathway that leads to distinct patterns of modification in foods. These new molecules are highly antigenic, and many important neoantigens found in cooked or stored foods are produced through Maillard reaction [33]. Therefore, the foundation of AGE during food processing through this mechanism of action and IgG, IgA and IgM antibodies produced against them can have a potent impact in tissue inflammation, a process linked to diverse biological settings such as diabetes, metabolic syndrome, renal failure and aging [34,35].

Elevation of antibodies against modified food antigens and their possible cross-reaction with AGE-HSA, AGE-Hb and ox-LDL suggest formation of high levels of glycated and lipoxidated proteins and peptides, including HbA1c [36,37].

RAGE, a receptor for AGE that has been known since 1992, induces activation of nuclear factor κB (NFκB) and converts long-lasting proinflammatory signals into cellular dysfunction, resulting in disease [38-40]. The binding of AGE to its receptor RAGE results in RAGE activation and the production of dysfunctioning proteins in tissues and in blood, and is strongly associated with a series of diseases from allergy and Alzheimers to rheuamtoid arthritis and urogenital disorders [34].

During the processing of food many lipids may become oxidized [30]. Auto-oxidized lipids could interact with various proteins and form new antigenic materials. For this reason, we measured ox-LDL antibodies in samples with or without antibodies aqgainst modified food antigens. IgG-ox-LDL antibody correlated with AGE-HSA, AGE-Hb and modified food antigens (Table 2). In addition to oxidation, LDL, like most plasma proteins, is also susceptible to AGE modification. AGE-modified food proteins are immunogenic, a property that has been used to great advantage for their detection in serum and localization in tissues [41-43]. Antibody formations against ox-LDL and AGE-LDL are able to combine with circulating ox-LDL or AGE-modified antigens and form soluble immune complexes that may contribute to inflammation and autoimmunity [44,45].

Finally, since the earlier studies it has been suggested that modification in food antigen results in formation of AGE, and that AGEs play a signifcant role in neuronal stress, thus exacerbating aging and neurodegenerative processes in the brain [13,18,21,29,32,35,45,46]. We measured antibodies against MBP in patients with high levels of antibodies against modified food antigens, AGE-HSA, AGE-Hb and ox-LDL. Results showed that the majority of samples with antibodies against modified proteins and lipoproteins also produced antibodies against MBP. These results further support the idea that formation of AGEs by modified food antigens not only results in antibody formation against dietary proteins and peptides but could also result in antibody production against self proteins, possibly contributing to inflammation, autoimmunity, aging, diabetes and neurodegeneration [34].

From the results presented here, we propose that the determination of allergy and sensitivity to food in the population could be improved by measuring IgE, IgG, IgA and IgM antibodies against both raw and processed food antigens. Further research is needed to examine just how much an elimination diet could contribute to the reduction of these autoantibodies and a concomitant improvement in the conditions of those suffering from autoimmunity, metabolic syndrome, aging and neurodegenerative disorders.

## Abbreviations

Ig: Immunoglobulin; AGE: advanced glycation end product; HSA: human serum albumin; Hb: haemoglobin; ox-LDL: oxidized low density lipoprotein; MBP: myelin basic protein; SDS: sodium dodecylsulfate; ELISA: enzyme-linked immunosorbent assay; TBS: tris-buffered saline; BSA: bovine serum albumin; PNPP: paranitrophenylphosphate; OD: optical density; EU: ELISA unit; mU: micro unit; RAGE: receptor for advanced glycation end product; NFκB: nuclear factor κB.

## Competing interests

AV is the Chief Executive Officer and Technical Director of Immunosciences Lab., Inc.
